# Integrated hybrid architecture of metal and biochar for high performance asymmetric supercapacitors

**DOI:** 10.1038/s41598-021-84979-z

**Published:** 2021-03-08

**Authors:** Omid Norouzi, S. E. M. Pourhosseini, Hamid Reza Naderi, Francesco Di Maria, Animesh Dutta

**Affiliations:** 1 School of Engineering, University of Guelph, Guelph, ON N1G2W1 Canada; 2 Institute of Chemistry and Technical Electrochemistry, Poznan University of Technology, 60-965 Poznan, Poland; 3 School of Chemistry, College of Science, University of Tehran, Tehran, Iran; 4 Department of Engineering, University of Perugia, Via G. Duranti 67, 06125 Perugia, Italy

**Keywords:** Supercapacitors, Composites, Bioenergy

## Abstract

Two state-of-the-art electrodes were successfully synthesized and used to assemble both symmetric and asymmetric type supercapacitors. 3DFAB was fabricated by direct pyrolysis of green macroalgae in the presence of NaOH. Possible NaOH activation mechanisms are proposed, which explains the formation of oxygen functional groups through quick penetration of OH- and NaOH into the vacancies. To obtain CoTLM, the tile-like architecture of cobalt oxides was introduced to the 3D interconnected functional algal biochar (3DFAB) by a simple one-pot hydrothermal method under mild conditions. For the symmetric supercapacitors, the maximum specific capacitance of RAB, 3DFAB, and CoTLM were 158, 296, and 445 F g^−1^ at the current density of 1 A g^−1^. Regarding cobalt-based asymmetric systems, the maximum capacitance for the 3DFAB//CoTLM was 411 F g^−1^. This asymmetric supercapacitor device also retained 100.9% of its initial capacitance after 4000 cycles at the current density of 4 A g^−1^. Unbuffered aqueous electrolyte and the unique morphological structure used in this study might catapult forward commercialization of such advanced energy storage devices.

## Introduction

Industrial and agricultural pollutants have significantly changed the level of nutrients, primarily nitrogen and phosphorus, in oceans, seas, and rivers^1^. These changes, directly or indirectly, cause damage to the environment on a global scale. Eutrophication, the excessive blooming of macroalgae, is a visible, alarming, and devastating phenomenon occurring as a result of such human activities. To mitigate the issue of eutrophication, many types of research have been conducted to evaluate the viability of using these harmful microalgae as a source of biofuel^2,3^. However, macroalgae biofuel production has not been fully commercialized due to the significant economic and technical challenges. To minimize waste and improve the circular economy's efficiency, biochar obtained as a byproduct of the thermochemical conversion of macroalgae could be further processed for versatile applications in energy conversion and storage sectors^4–8^. The best-known example is the application of biochar as a promising alternative to its commercial competitors in supercapacitors due to their apparent advantages such as low cost, accessibility, reduced environmental impact, and good stability^9,10^.

Algal biochar has a unique advantage over agricultural wastes in the way that macroalgae undergoes self-activation and nitrogen self-doping during the thermal process due to the abundanceof potassium (K), calcium (Ca), magnesium (Mg), sodium (Na), and nitrogen (N) that exist in the algae structure^11^. However, there is still much work to be done in reaching the desired electrochemical properties in supercapacitors by rationally integrating the physical and chemical modification methods. Algal biochar needs to be further be processed to achieve a highly improved EDLC and pesocapacitance performance. Most state-of-the-art modified biochar composites with excellent capacitive performance have been reviewed comprehensively by Norouzi et al.^12^. The simplest and most popular chemical surface modification is NaOH or/and KOH activators' use before or after the thermal process. For example, Hu et al. successfully synthesized porous particulate activated carbons from low sulfonate content alkaline lignin by hydrothermal carbonization in the presence of NaOH and NaOH modifiers. The synthesized material showed a hierarchical structure with improved S_BET,_ functional groups, and EDLC performance^13^. In addition to chemical modification, it is beneficial to simultaneously enhance the sample's pesocapacitance by introducing pseudocapacitive materials into the biochar structure. To this end, transition metal oxides or hydroxides are usually embedded in the porous structure of the surface-modified biochar^14–17^. Among the available pseudocapacitive materials, cobalt hydroxides or oxides are favorable candidates for application in electrochemical capacitors due to their low cost, great reversibility, high conductivity, multiple oxidation states, and high specific capacitance. Cobalt oxide (Co_3_O_4_) has an extremely high theoretical specific capacitance of up 3560 F g^−1^, which has been recently receiving more attention within the electrochemistry research groups^18,19^.

Apart from designing a hybrid biochar electrode, selecting two dissimilar electrode materials with well-separated potential windows, called Asymmetric supercapacitors (ASCs), plays a vital role in reaching higher energy density, power density, and cycle life^20,21^. In this study, a cost-effective ACSs was fabricated using a new interconnected tile-like microstructure containing cobalt oxide particles, referred to as CoTLM, and functional algal biochar composed of a 3D interconnected mesopores network (3DFAB). The CoTLM composites were synthesized by impregnation of Co(NO_3_)0.6H_2_O on the surface of algal biochar under hydrothermal carbonization. FAB was prepared by direct pyrolysis using green macroalgae as the carbon precursor and NaOH as the activator.

## Results and discussions

**Figure 1:**
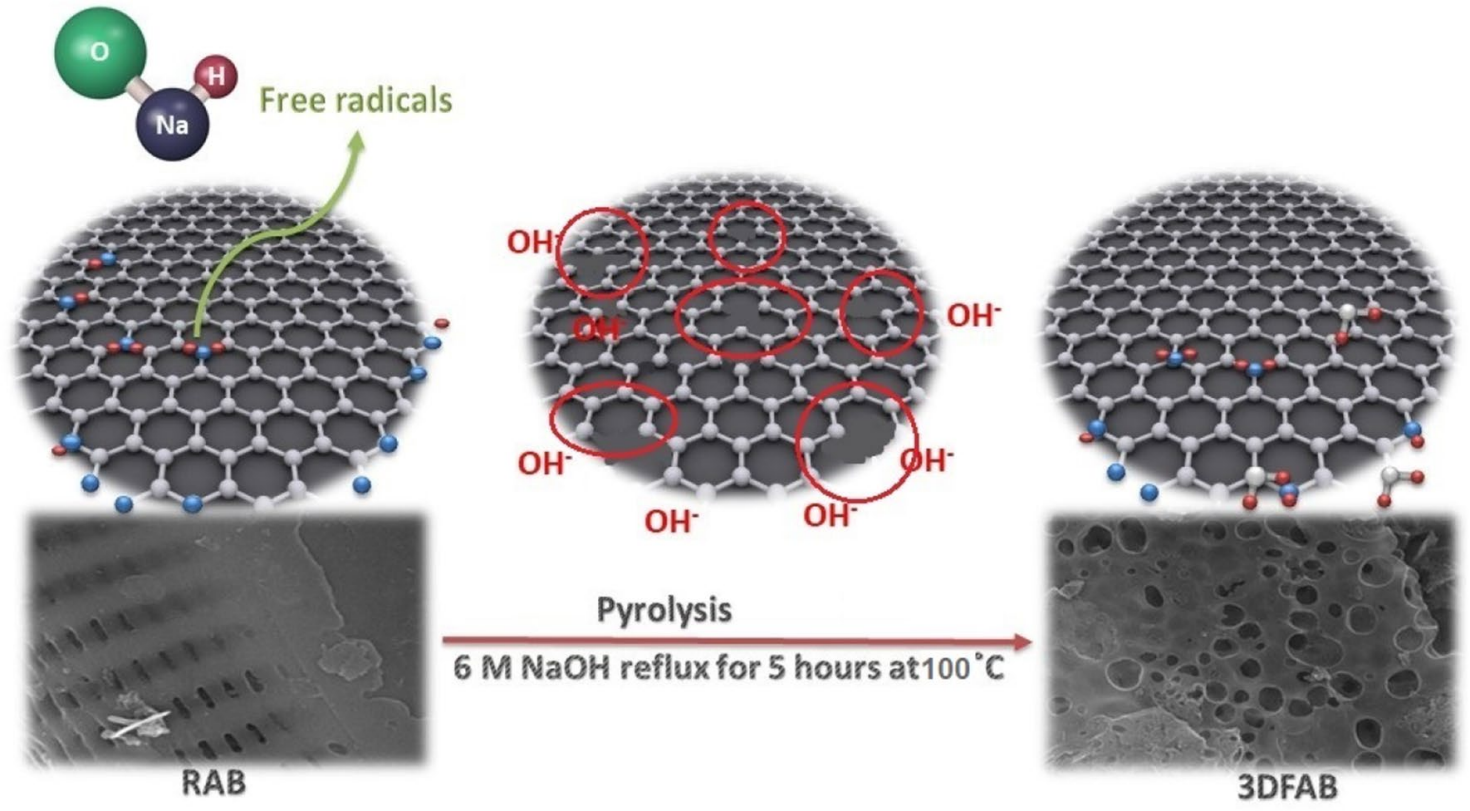
Possible mechanism of NaOH activation for the synthesis of 3FAB.

**Figure 2:**
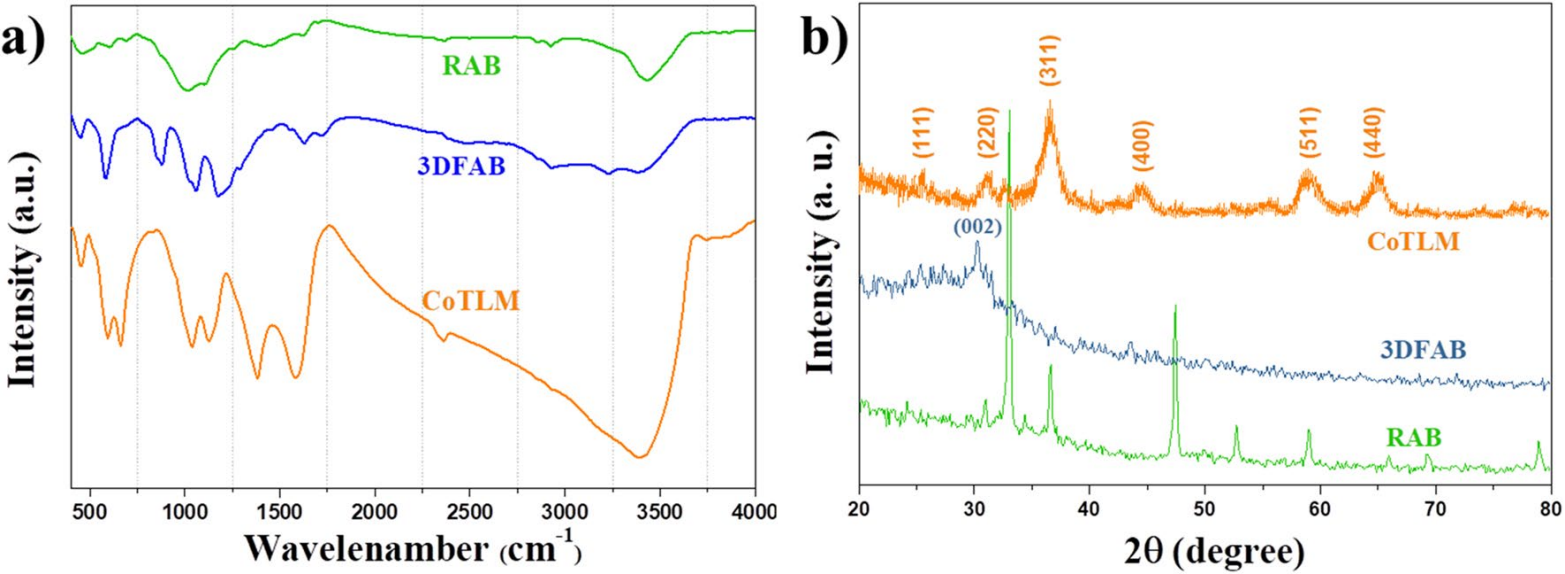
FT-IR spectra (**a**) and XRD patterns (**b**) of RAB, 3DFAB, CoTLM.

FTIR analysis was carried out to indicate the rate and degree of decomposition during the synthesis based on functional groups. All three samples have similar FTIR spectra but at different intensities (Fig. 2a). The spectra of glycosyl-units of cellulose are detected in the range of 1150–1070 cm^−1^ due to the stretching vibrations of CH–, OH–, and CH_2_– groups^22^. The strongest peaks at these ranges are observed in the CoTLM spectrum, showing the more intense glycosidic bond-breaking reactions. The FT-IR spectrum of CoTLM exhibits a broader peak at 2000–3400 cm^−1^, which can be attributed to its higher hydrophilic and conductive nature. Peaks at 1457, 1592, and 1710 cm^−1^ are assigned to the ester and carboxylic acid functional groups in cellulose-based polysaccharides^6,22–25^. These peaks have been pronounced in 3DFAB, as compared to the RAB, as the result of a series of reactions, which have been shown in Eqs. (1–6) and Fig. 1. During the pyrolysis process, NaOH vigorously reacts with carbonyl, hydroxyl, carboxyl, ether, and ester functional groups to produce free radicals as well as a number of vacancies. At the same time, many vacancies are created due to the NaOH reaction with the C–C and C–H groups. Many oxygen functional groups are then formed by quick penetration of OH- and NaOH into the vacancies. Finally, NaOH reacts with oxygen functional groups over in carbon fragments at 400–700 °C, which can produce K_2_CO_3_ (Eqs. 1–6).

According to the literature, most of the acidic functional groups attached to the surface of 3DFAB should react with cobalt ions (CO^2+^ and CO^3+^) during HTC to produce water and cobalt nanoparticles (Eqs. 7 and 8). Thus, a lower intensity of -O containing groups was excepted but was not found. This could be due to the improved hydrolysis reactions in HTC by which polysaccharides as macro-intermediates are produced from the unreacted cellulose remaining after the pyrolysis.

1 $$ {\text{6NaOH }} + {\text{ 2C}} \to {\text{2K}}_{{2}} {\text{CO}}_{{3}} + {\text{ 2Na }} + {\text{ 3H}}_{{2}} $$

2 $$ {\text{NaOH }} + \, ( - {\text{COOH}})/( - {\text{O}} - {\text{C }} = {\text{ O}}) \, \to {\text{ Na}}_{{2}} {\text{CO}}_{{3}} + {\text{ Na }} + {\text{ H}}_{{2}} + {\text{ CO}}_{{2}} $$

3 $$ {\text{NaOH }} + \, ( - {\text{C }} = {\text{ O}})/({\text{C}} - {\text{O}} - {\text{C}}) \, \to {\text{ Na}}_{{2}} {\text{CO}}_{{3}} + {\text{ Na }} + {\text{ H}}_{{2}} + {\text{ CO}} $$

4 $$ {\text{NaOH }} + \, ( - {\text{O}} - {\text{CH}}_{{3}} ) \, \to {\text{ Na}}_{{2}} {\text{CO}}_{{3}} + {\text{ Na }} + {\text{ H}}_{{2}} + {\text{ CH}}_{{4}} $$

5 $$ {\text{NaOH }} + \, ({\text{C}} - {\text{OH}}) \, \to {\text{ Na}}_{{2}} {\text{CO}}_{{3}} + {\text{ Na }} + {\text{ H}}_{{2}} {\text{O }} + {\text{ H}}_{{2}} $$

6 $$ {\text{NaOH }} + \, ({\text{C}} - {\text{H}}) \, \to {\text{ Na}}_{{2}} {\text{CO}}_{{3}} + {\text{ Na }} + {\text{ H}}_{{2}} $$

7 $$ {\text{Co}}^{{{2} + }} - {\text{ e }} \to {\text{ Co}}^{{{3} + }} $$

8 $$ {\text{2Co}}^{{{3} + }} + {\text{ Co}}^{{{2} + }} + {\text{ 8OH}}^{{{1} - }} + {\text{ 3DFAB }} \to {\text{ CoTLM }} + {\text{ 4H}}_{{2}} {\text{O}} $$

Figure 2b shows XRD patterns of RAB, 3DFAB, and CoTLM. The crystalline region of cellulose found at 2θ about 22.45° and 34.25°^26^. Since the experiments were performed in a harsher synthetic condition for modified samples, strong peaks assigned to cellulose lost their intensity. In other words, for 3DFAB, and CoTLM, an amorphous structure, and disordered graphitic (002) plane was found at Bragg's angle between 20° and 30° due to the crystalline-to-amorphous transformation of cellulose under intensive thermal conditions^22^. Other sharp peaks at 2θ = 30.34°, 32.4°, 36.99°, 40.41°, 44.00°, 48.47°, 49.43°, 58.38°, and 61.64° are related to one of the calcium-based crystalline structures. Three identified phases are CCaO_3_, Ca_6.00_C_3.00_O_18.00_, Ca_6.00_C_6.00_O_18.00_, and Ca_6.00_C_6.00_O_18.00_. In the case of CoTLM, peaks at 19.0°, 31.2°, 36.8°, 38.5°, 44.8°, 55.6°, 59.3°, 65.2°, and 77.3° confirm the crystalline structure of Co_3_O_4_, which is in accordance with the Joint Committee on Powder Diffraction Standard (JCPDS No. 00-042-1467)^27–29^.

**Figure 3:**
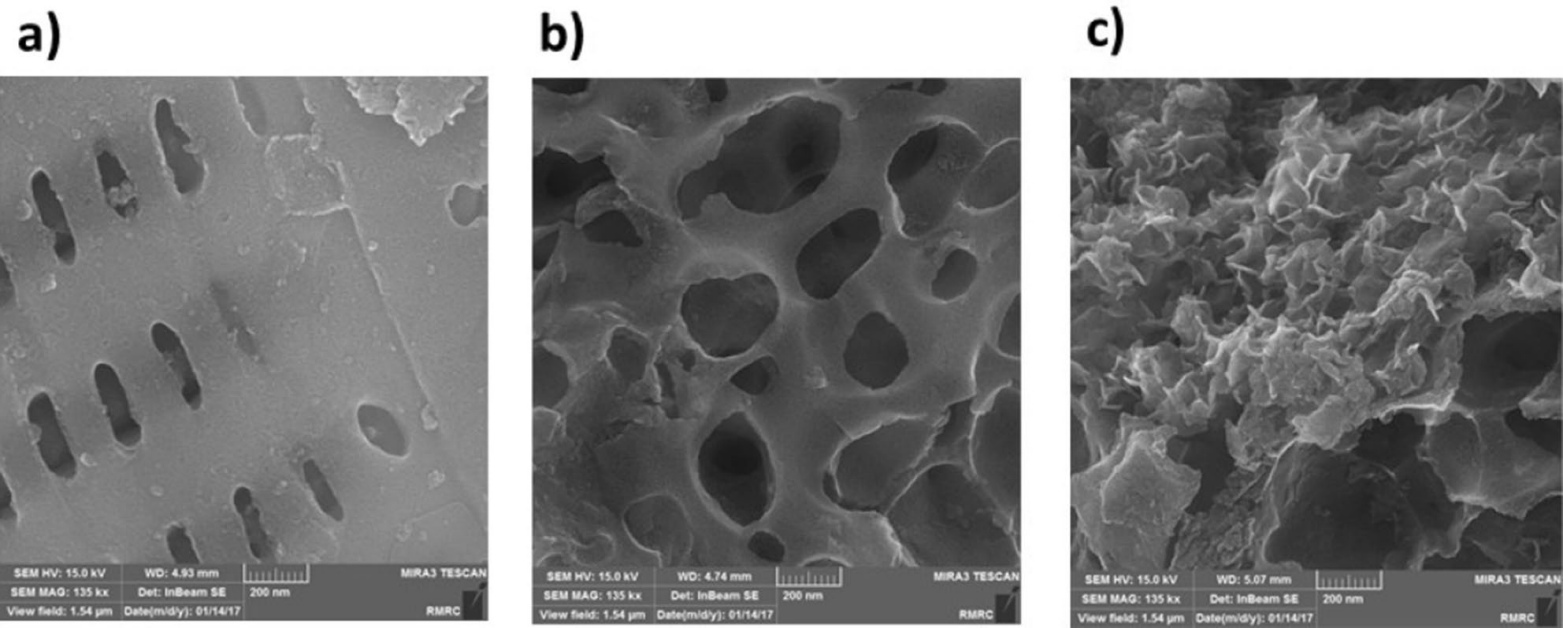
FESEM images of the RAB (**a**), 3DFAB (**b**), and CoTLM (**c**).

Figure 3a shows that the raw biochar derived from green macroalgae has an olive-shaped morphology with hollow macropores, which are evenly spread with an average diameter of approximately 300 nm wide and 80 nm long. One interesting finding is that RAB's olive-shaped morphology is completely transformed into the circular-shaped structure in which pores are interconnected in the three-dimensional structure of 3DFAB (Fig. 3b). Figure 1 and Eqs. (1–6) explain the reason behind this morphology change through possible chemical reaction pathway of NaOH activation during biomass pyrolysis. These reactions resulted in an advanced morphology due to the release of large amounts of gaseous products. The 3D interconnected functionalized mesopores network can improve the accessible inner/outer surfaces and facilitate the formation of Co_3_O_4_ via improved impregnation of cobalt salt solution using functional groups^11^. The synthesized CoTLM possesses multilayered structures made from hierarchical nanosheets, making it an advanced material with a tile-like microstructure (see Fig. 3c). This architecture has already been observed by Shurui Liu et al.^30^. The unique hierarchical architecture of CoTLM provides a continuous pathway for electrons and shorten diffusion pathways for ions, thereby making it a great candidate for charge storage purposes. We have provided our readers with some more FESEM images in supporting information (Figure S1) to further analyze the morphological structure.

Furthermore, after applying chemical and/or physical modifications on the green algae, elemental surface distribution in 3DFAB and CoTLM was changed significantly, verified by EDS analyses. The quantitative results of the EDS are given in Table 1. Approximately 21% of the RAB's surface is found to be covered by alkali and alkaline earth metals (AAEMs) such as sodium, potassium, calcium, and magnesium. These elements belong to the ash portion of the algae. The RAB contains 15% of sulfur and silica, which are considered essential micronutrients for the normal growth of algae^31^. Oxygen content in 3DFAB has more than doubled due to the elimination of AAEMs by NaOH treatment and acid reflux. However, improved oxygen contents in CoTLM are mostly related to the attachment of oxygen functional groups produced under the hydrothermal treatment. Based on surface oxygen contents, we conclude that part of the CoTLM surface was developed by oxygen functional groups, making them more polar and hydrophilic than the original biochar derived from green algae. During the HTC in the presence of Co(NO_3_)0.6H_2_O, cobalt particles were efficiently dispersed over the surface and caused improved oxidation–reduction reactions (pseudocapacitance) and higher electrical conductivity of the sample.

**Table 1:** EDS analyses of RAB, 3DFAB, and CoTLM prepared from green macroalgae.

Element W%	C	O	N	Na	Mg	Al	Si	S	K	Ca	Fe	Co
RAB	41.12	22.00	–	1.21	1.24	0.75	2.32	13.0	2.92	15.3	0.15	–
3DFAB	36.47	55.78	4.00	–	–	–	1.92	1.83	–	–	–	–
CoTLM	22.69	31.18	–	–	–	–	1.54	2.52	–	–	2.23	39.84

**Figure 4:**
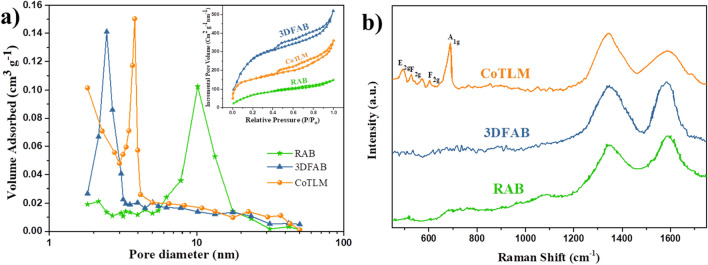
N_2_ adsorption/desorption isotherm and pore size distributions (BJH) of the RAB, 3DFAB, CoTLM (**a**), Raman analyses (**b**).

Figure 4 displays the N_2_ adsorption–desorption isotherms and BJH pore diameters of RAB, 3DFAB, CoTLM samples. All the N_2_ adsorption–desorption isotherms exhibited the same kinetic reaction with a typical IV hysteresis loop at a relative pressure between 0.45 and 0.95, which confirms the hierarchical porous structure of samples^32–34^. The majority of RAB pore volume is in the range of 0.10 cm^2^ g^−1^ nm^−1^. However, the pore volume in 3DFAB and CoTLM samples fluctuates between 0.10 and 0.14 cm^2^ g^−1^ nm^−1^, owing to the 3D architecture that occurred after the treatment. BET surface area of RAB, 3DFAB, and CoTLM were 243, 1020, and 605 m^2^ g^−1^, respectively. The characterization results reveal that both 3DFAB and CoTLM have a higher surface area, larger pore volume, and smaller pore diameter compared to the original biochar. The higher surface area and interconnected 3D pore network in the 3DFAB could be due to reaction 1 (Eq. 1) in which carbon reacts vigorously with sodium hydroxide to form Na_2_O_(s)_ along with hydrogen and carbon dioxide gas. Considering the superior textural properties of 3DFAB, it might be a highly promising start material for accommodating conductive materials in nanoscale. Herein, CoTLM was obtained through the dispersion of cobalt oxide particles within the interior of the interconnected 3D pore network of 3DFAB. CoTLM can not only facilitate charge transfer and ion diffusion but also take advantage of the pseudocapacitive nature of Co_3_O_4_ nanoparticles^35^.

Raman spectroscopy was performed to analyze further the carbon structure of RAB, 3DFAB, and CoTLM. According to the Raman spectra of samples (Fig. 4b), two main peaks were recorded at around 1345 cm^−1^ (D band) and 1570 cm^−1^ (G band), which are ascribed to the turbostratic and ideal graphitic carbon structure. I_D_/I_G_ can reflect the disordered degree in the modified samples. The I_D_/I_G_ ratio for RAB, 3DFAB and CoTLM was 0.91, 1.05, and 1.65, respectively. Raman spectrum of CoTLM shows some characteristic peaks at 486, 529, 608, and 678 cm^−1^, which are related to the vibrational modes of E_g_, ${\text{F}}_{{2{\text{g}}}}$, ${\text{F}}_{{2{\text{g}}}}$, and A_1g_, respectively^36^.

### Electrochemical performance

**Figure 5:**
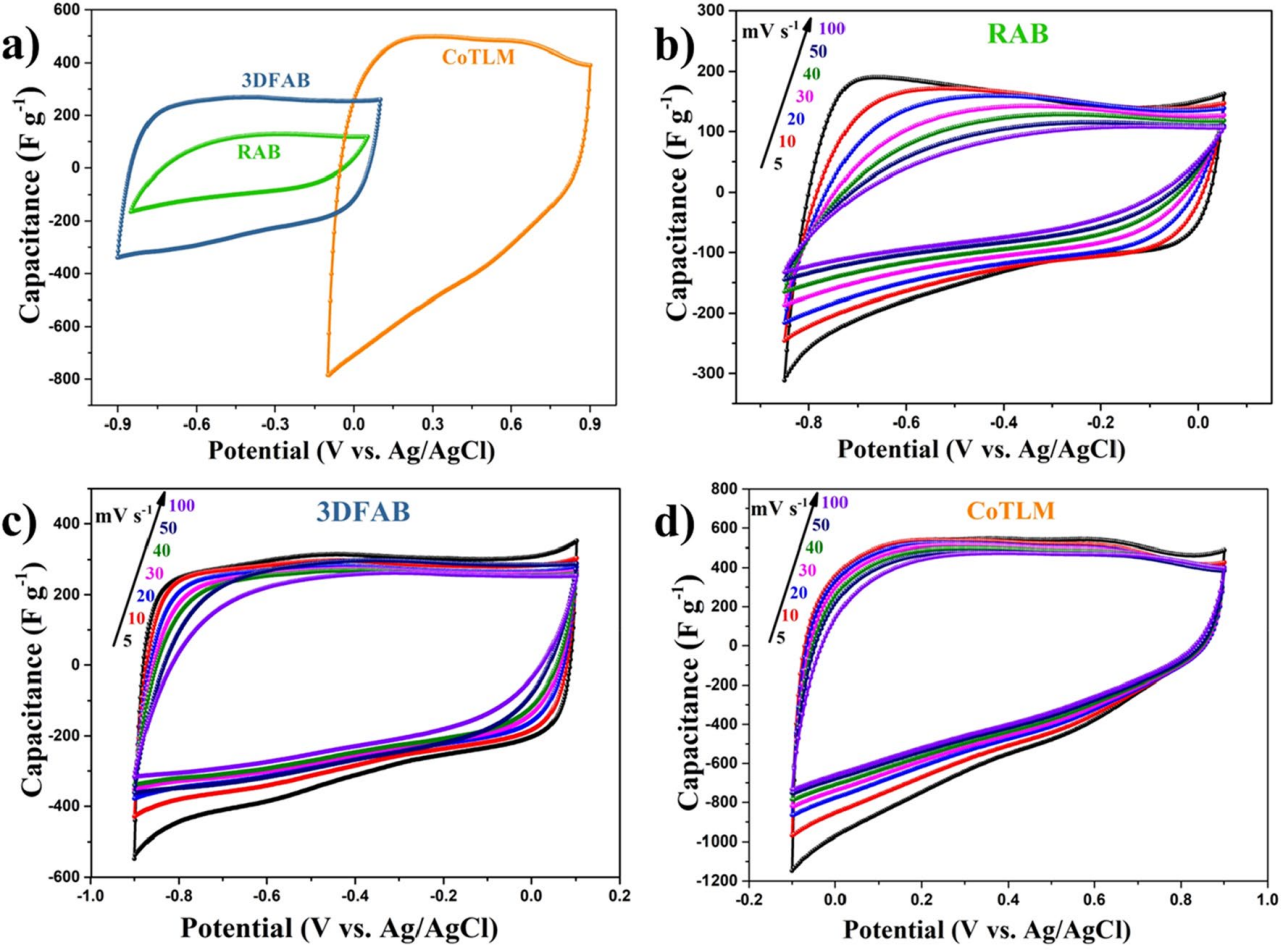
Electrochemical performance of the RAB, 3DFAB, and CoTLM electrodes. Cyclic voltammograms at 50 mV s^−1^ (**a**); cyclic voltammograms RAB, 3DFAB, and CoTLM at increasing rates from 5 to 100 mV s^−1^ (**b**–**d**).

*CV measurements* Figure 5a gives typical cyclic voltammetry (CV) curves of the RAB, 3DFAB, and CoTLM at a scan rate of 50 mV s^−1^. The CV of RAB, 3DFAB, CoTLM were in the range of − 0.85 to 0.05 V, − 0.9 to 0.1 V, and − 0.1 to 0.9 V (vs. Ag/AgCl), respectively. The slight shift toward higher voltages in the redox potential of CoTLM, resulting from the unique 3D morphology and structure of the electrode material, shows the more polar nature of CoTLM^30^. Figure 5b–d shows typical CV curves of all three state of the art electrodes at various scan rates of 5–100 mV S^−1^. They all have shown a quasi-rectangular shape even at the highest applied scan rate (100 mV S^−1^), which is an indication of their excellent charge transfer capability^20,37,38^. The closest shape to an ideal rectangular can be found in 3DFAB (Fig. 5c), which might result from its ordered interconnected 3D pore network and superior surface area. The slower scan rate gives the electrolyte ions sufficient migration time to penetrate better within the interior of the porous carbons. At higher scan rates, electrolyte ions can only accumulate on the outer surface of samples^21,39^. The potential window of CoTLM is in the positive potential (vs. Ag/AgCl); while the potential window of 3DFAB is in the negative potential (vs. Ag/AgCl). The stable shape of CV in the high scan rate along with negative potential window make 3DFAB a good choice for negative electrodes. For the same reason above, CoTLM is suitable for positive electrodes in a full system. In the asymmetric system the positive electrodes is able to store energy with both faradic and non-faradic mechanism.

**Figure 6:**
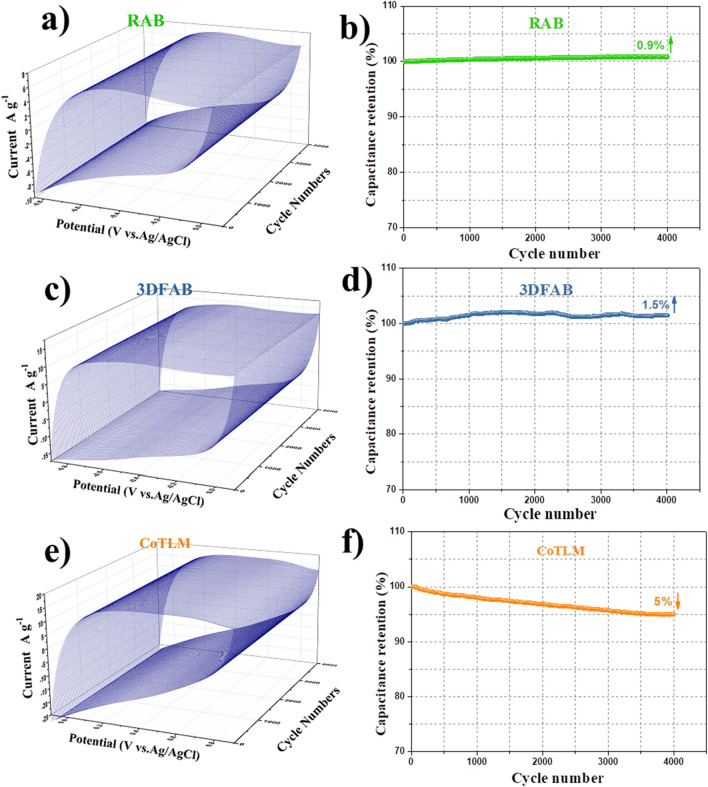
3D-CCV curves of the RAB, 3DFAB, and CoTLM electrodes measured at a scan rate of 50 mV s^−1^ (**a**–**c**), variation of the specific capacitance of the RAB, 3DFAB, and CoTLM electrodes with CCV method as a function of the number of cycles at 50 mV s^−1^ (**d**–**f**).

*CCV measurements* Figure 6 shows the RAB's cyclic performance, 3DFAB, CoTLM under 4000 cycles at the scan rate of 50 mV s^−1^. For the CoTLM electrode, 5% of the capacitance is dropped due to the volume alternation in the electrode material caused by electrolyte intercalation and deintercalation reactions during long potential cycling^40^. However, two other non-metal-modified electrodes, RAB and 3DFAB, exhibited superior cycling stability with 100.9% and 101.5% retention of their initial capacitance after 4000 cycles, respectively. This phenomenon has already been reported in the literature as the gradual activation of the surface with increasing the number of cycles. The results indicate that the electrochemical stability of the 3DFAB is higher than that of others, which makes it a suitable electrical double layer capacitor and a great candidate for accommodating pseudocapacitors.

**Figure 7:**
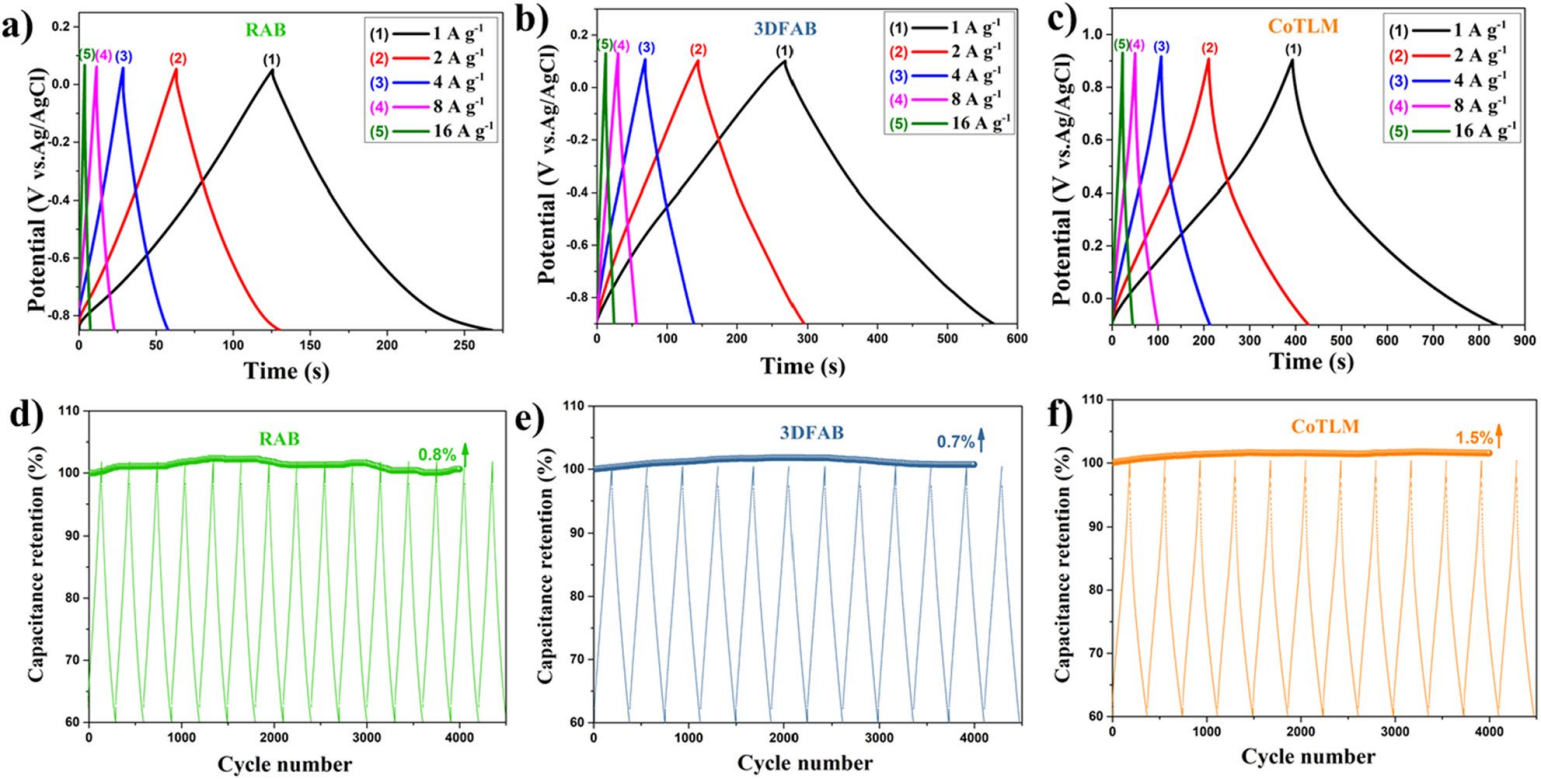
GCD of RAB, 3DFAB, and CoTLM at the current densities rates from 1 to 16 A g^−1^ (**a**–**c**); and cyclic performance of the RAB, 3DFAB, and CoTLM electrodes at the current density of 4 A g^−1^ (**d**–**f**).

GCD measurements. GCD curves of RAB, 3DFAB, and CoTLM are recorded and shown in Fig. 7a–c. The measurements were performed at current densities ranging from 1 to 16 A g^−1^. They all have symmetrical triangular shapes with any notable IR drop, suggesting their excellent charging-discharging behavior^41,42^. The maximum SC for RAB, 3DFAB, and CoTLM at the current density of 1 A g^−1^ were 158, 296, and 445 F g^−1^, respectively. The triangular, linear, symmetric, and very sharp curves reflects their reversible behavior, high coulombic efficiency, and ideal capacitor performance.

Capacitance retention as a function of cycle number, at the current density of 1 A g^−1^, is plotted for RAB, 3DFAB, and CoTLM in Fig. 7d–f, respectively. As shown, three electrodes have a nearly identical capacitance drop after 4000 cycles. In all samples, the capacitance is promoted around 1%, indicating their superior cyclic performance. This slight increase in capacitance after 4000 cycles is due to the improved access of electrolyte ions to the new activated sites at higher cycles^11^.

To better demonstrate the synthesized sample's superior performance, the literature is comprehensively reviewed, and the results are presented in Table 2. As for the cyclic stability, CoTLM's stability slightly increased by 1.5% after 4 k cycles, which is significantly better than its counterparts presented in the table. This phenomenon could be due to the unique 3D morphological structure of CoTLM facilitating both conductivity and ion diffusion. Among those composites that nano-carbons have integrated with cobalt nanoparticles, 'reduced graphene oxide/cobalt oxide composites' and 'Co_3_O_4_/carbon aerogel microbead' exhibited a capacitance of 291 and 350 F g^−1^, which are still lower than our reported SC (445 F g^−1^ ) tested at the same measurement condition. When it comes to the electrolyte, the milder condition you use, the higher possibility for its commercialization exists. For the recent cobalt-based composites, typical basic aqueous solutions such as NaOH and KOH with various concentrations, ranging from 1 to 6 M have been used. However, in our study, KCl was introduced to the system, which is an unbuffered aqueous electrolyte. Based on the Nernst equation, such neutral electrolytes enable us to work on broader potential windows under a non-corrosive condition^43^.

**Table 2:** Electrochemical results derived from cobalt-based electrodes.

Sample	Electrolyte	Specific capacitance (F g^−1^)	Measurement condition	Cycling stability	References
Graphene/Co_3_O_4_ composites	1 M NaOH	433	0.5 A g^−1^	97.1% after 1000 cycles	^44^
Cobalt oxide/multi-walled carbon nanotube composites	2 M KOH	418	0.625 A g^−1^	91% after 2000 cycles	^45^
Carbon blacks filler/Co_3_O_4_/graphene nanosheets	1 M KOH	694	2 A g^−1^	91.9 after 3000 cycles	^46^
Multi-walled carbon nanotubes/Co_3_O_4_ nanocomposites	1 M KOH	201	10 mV s^−1^	–	^47^
Reduced graphene oxide/cobalt oxide composites	6 M KOH	291	1 A g^−1^	90% after 1000 cycles	^48^
Co_3_O_4_/carbon aerogel microbead	6 M KOH	350	1 A g^−1^	90% after 5000 cycles	^49^
Reduced graphene oxide/Co_3_O_4_ composite	2 M KOH	472	2 mV s^−1^	95.6% after 1000 cycles	^50^
Graphene nanosheet/Co_3_O_4_ composite	6 M KOH	243.2	10 mV s^−1^	95.6% after 2000 cycles	^51^
Cobalt hydroxide nanosheets on carbon nanotubes/carbon paper	6 M KOH	1083	0.83 A g^−1^	82.5 after 1000 cycles	^25^
CoTLM	3 M KCl	445	1 A g^−1^	101.5% after 4000 cycles	This work

**Figure 8:**
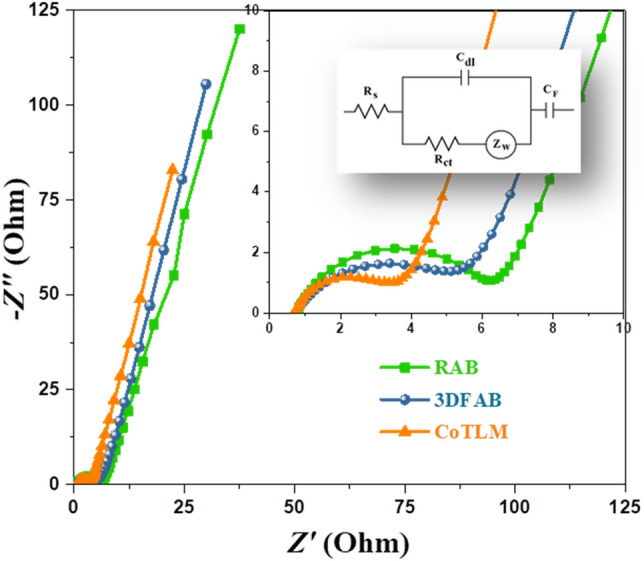
Nyquist plots of the RAB, 3DFAB, and CoTLM electrodes.

EIS measurements. Charge transfer kinetics and ion diffusion rates were studied by EIS analysis and the reults are given in Table 3. The Nyquist plots of RAB, 3DFAB, and CoTLM are shown in Fig. 8. EIS measurements were conducted at the frequency range from 0.01 to 100 kHz at a potential of about -0.45 V with an alternate amplitude voltage of 5 mV. The equivalent circuit can fit EIS data. Charge transfer resistance (R_ct_) and ohmic resistance (R_s_) were obtained by calculating the semicircle diameter in the high-frequency regions and intercept of the real axis in the Nyquist diagrams, respectively. The CoTLM electrode shows a lower R_ct_ (2.6 Ω) than that of RAB (5.1 Ω) and 3DFAB (3.9 Ω), confirming its remarkable electrical conductivity. Moreover, a higher slope is recorded for CoTLM electrode, revealing lower R_s_ (0.71 Ω) in this electrode as compared to RAB (0.84 Ω) and 3DFAB (0.81 Ω). The Warburg resistance, symbol Z_w_, is the straight line at low-frequency regions. These lines show the variations in ion diffusion path lengths. As seen in Table 2, RAB, 3DFAB, and CoTLM have a Z_w_ of 0.11, 0.13, 0.21 Ω, respectively. We can conclude that modification of RAB resulted in shorter ion diffusion paths and fewer barriers to ion movement^52^.

**Table 3:** Calculated values of RS, CPE, Rct, ZW, and CF through CNLS fitting of the experimental impedance spectra based upon the proposed equivalent circuit.

	RAB	3DFAB	CoTLM
R_S_ (mOhm)	0.84	0.78	0.71
C_dl_ (mF)	0.5	1.5	1.9
R_ct_ (Ohm)	5.1	4.3	2.6
Z_W_ (MMho)	0.11	0.14	0.18
C_F_ (mF)	130	150	250

### Electrochemical performances of the and 3DFAB//CoTLM ASC devices

**Figure 9:**
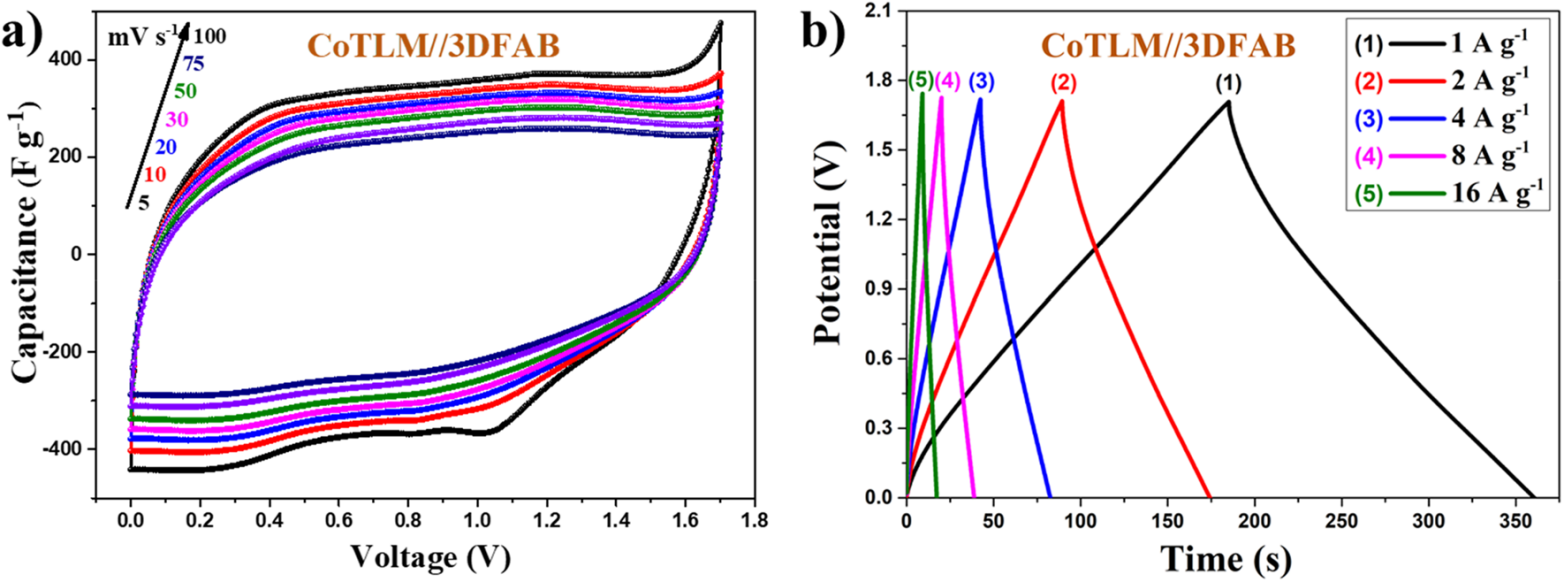
CV curves at different scan rates of 3DFAB//CoTLM (**a**), and charge/discharge curves under different current densities of 3DFAB//CoTLM (**b**).

For the ASC, 3DFAB has been considered as a positive faradic electrode due to its superior surface area and suitable potential window. On the other hand, CoTLM showed a stable and suitable potential window in the region of negative chosen electrodes. The working potential range of 3DFAB was − 0.9 to 0 V, while that of CoTLM was − 0.1 to 0.9 V. Thus, this two-electrode combination's cell voltage has extended up to 1.7 V, which is significantly higher than that obtained in symmetric type supercapacitors. CV diagrams and charge/discharge curves of 3DFAB//CoTLM devices are shown in Fig. 9a,b. All the CV curves of the ASC devices remained unchanged, indicating that electrons and ions can quickly move within the pore structure of the electrodes even at 100 mV s^−1^, suggesting the ASC cell possesses high power capability. Simultaneously, charge–discharge curves have almost retained their symmetrical shape at different current densities, suggesting their high coulombic efficiency and good electrochemical reversibility of these two asymmetric systems.

**Figure 10:**
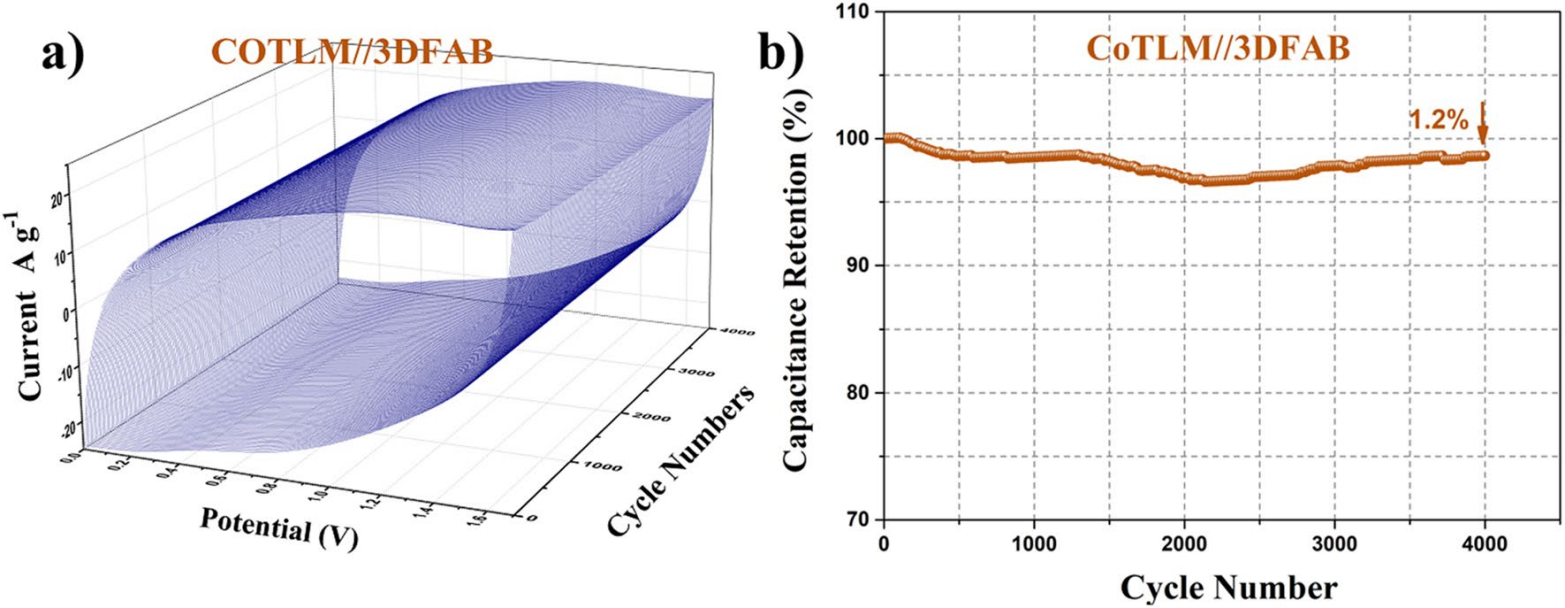
3DFAB//CoTLM asymmetric systems measured at a scan rate of 50 mV s^−1^ (**a**), variation of the specific capacitance of the 3DFAB//CoTLM cell with CCV method as a function of the number of cycles at 50 mV s^−1^ (**b**).

Figure 10a shows 3D-CCV curves of 3DFAB//CoTLM electrode measured at a scan rate of 50 mV s^−1^ for 4000 cycles. As shown in Fig. 10b, the asymmetric systems showed superior cyclic stability and during the cycling, the additional peaks did not appear. Up to 98.8% of the capacitance was retained for 3DFAB//CoTLM; the results were further processed into a Capacitance-Cycle number diagram to clarify the above statements.

**Figure 11:**
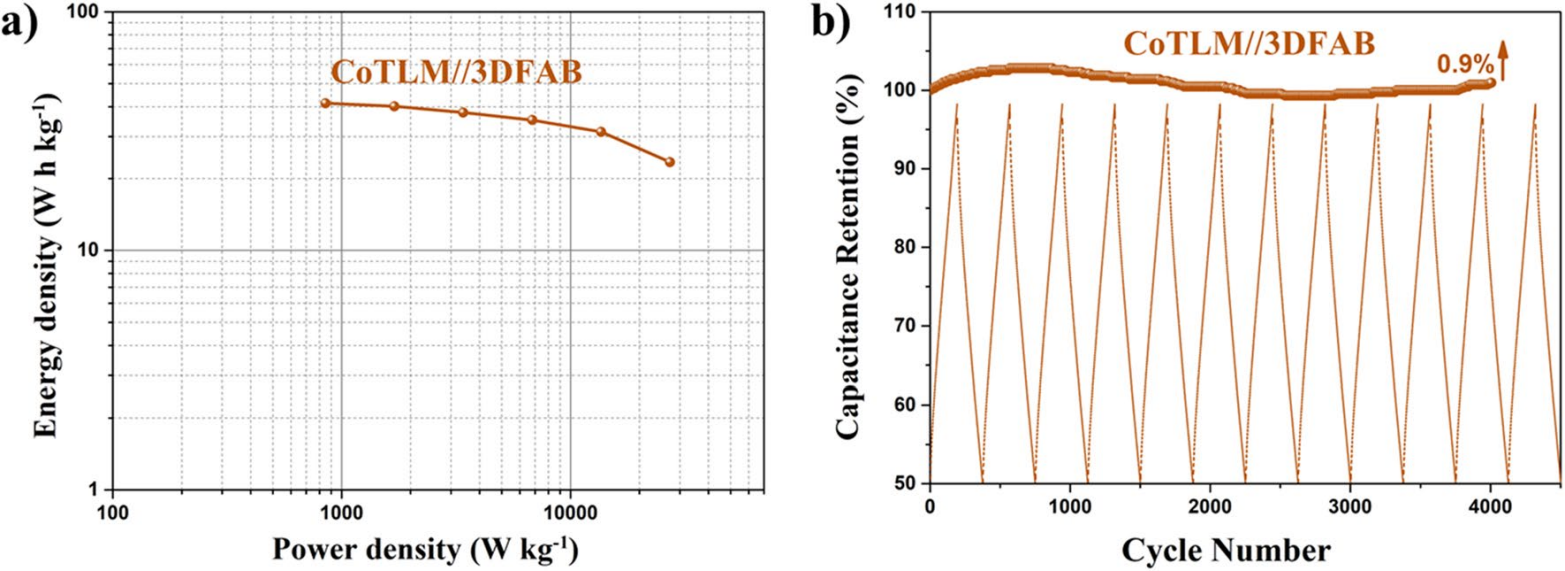
Cycle performance of 3DFAB//CoTLM electrode at the current density of 4 A g^−1^ (**a**), Ragone plot of 3DFAB//CoTLM (**b**).

To further support the above results, the cycle performance of 3DFAB//CoTLM electrode at the current density of 4 A g^−1^ is shown in Fig. 11a. Notably, 3DFAB//CoTLM electrode retained 100.9% of its initial capacitance after 4000 cycles at the current density of 4 A g^−1^. Although the capacity decay after long-term cycling for asymmetric cobalt-based electrodes has been observed previously, the reasons have remained somewhat uncertain. There are three main hypotheses for such phenomenon: i) slight changes in ternary oxides and/or hydroxide are caused by intensive reaction with KCl, and ii) damage in the morphology of tile-like Microstructure with a 3D architecture was due to the long-term cycling test.

Energy density and power density are two main factors that should be considered in scaling up the ACS devices. Figure 11b shows the Ragone plots of the 3DFAB//CoTLM electrode. The energy density acquired from the 3DFAB//CoTLM ASC device is 54.44 Wh kg^−1^ with a power density of 800 W kg^−1^, which is significantly larger than those reported for cobalt and iron-based composite ASCs^30^.

## Experimental section

**Figure 12:**
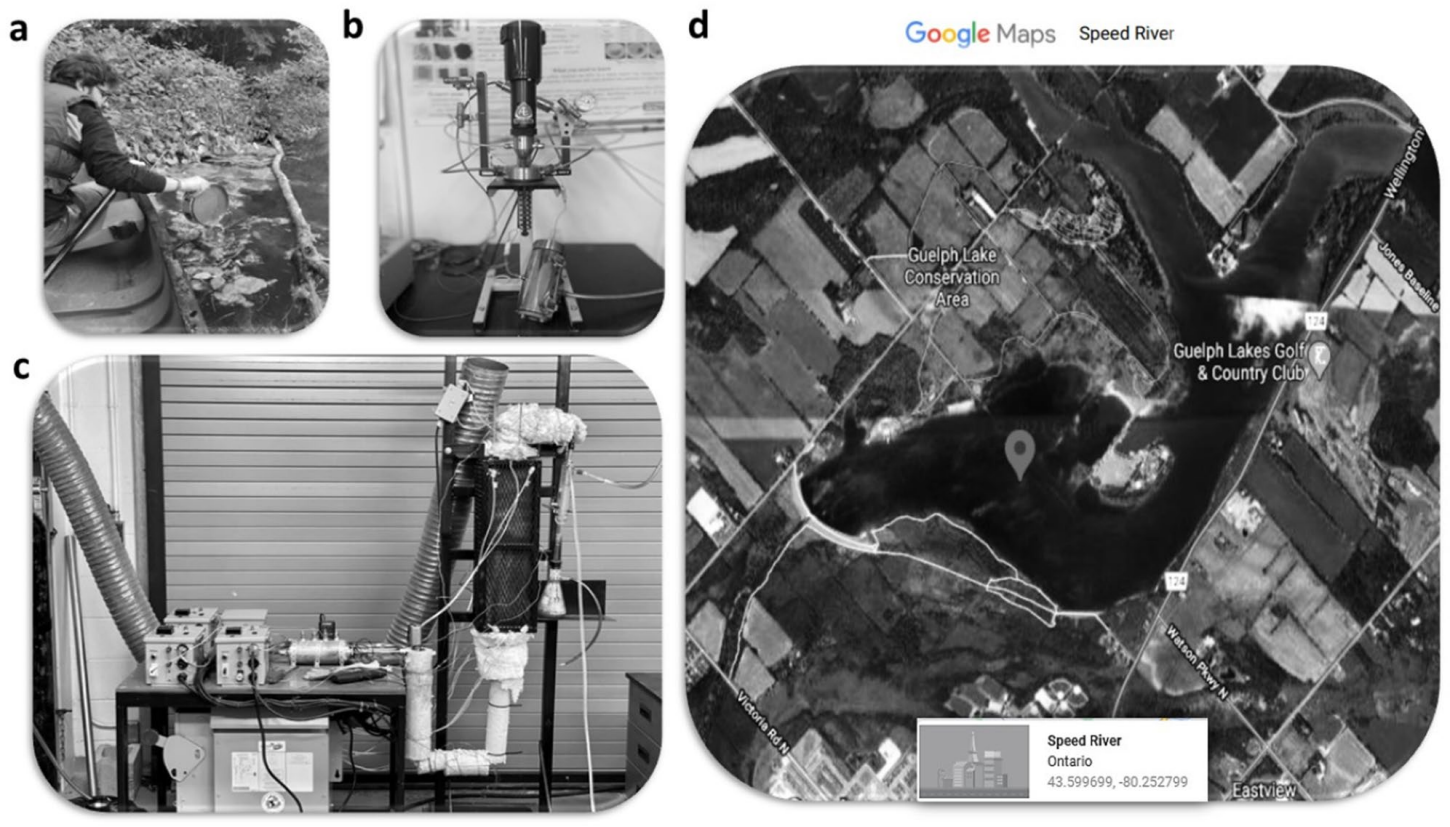
(**a**) physical location of sampling, schematics of the lab-scale experimental set up of the slow pyrolysis (**b**) and hydrothermal processes (**c**), and Satellite image (**d**).

Green macroalgae wastes (*Cladophora glomerata*) were collected by hand from different locations of the Speed River, Guelph, Ontario, Canada, during the months of June and July 2019 (see Fig. 12a). The exact sampling locations are shown in Fig. 12d. The algae were carefully washed with deionized water to remove sand, salt, and other contaminants attached to their surface. Afterward, any surplus of water was drained, and the samples were then dried at 105 °C in a furnace overnight. The dried algae were then ground and mixed to ensure the batch's uniform consistency and composition. RAB was prepared using a macro TGA at Bio-Renewable Innovation Lab, University of Guelph, Canada (Fig. 12c) and was further processed via HTL process (Fig. 12b). Five grams of green macroalgae (sieved into the particle size < 150 µm in diameter) were placed into a reactor consisting of a stainless-steel tube of 175 mm height and 15 mm and then the nitrogen gas purged the system before starting the reaction. Afterward, the purged reactor was inserted inside a Muffle Furnace (Model F48055-60, USA) to heat the pyrolysis reactor. The experiments were performed at a heating rate of 15 °C/min, reaching a temperature of 700 °C. The k-type thermocouple was connected to a data-logger to visualize and record the temperature profile on the computer continuously. RAB was utilized as a start material to synthesize3DFAB and CoTLM.

### Synthesis of 3DFAB

Four grams of green macroalgae were mixed with 6 M NaOH and refluxed for 5 h at 100 °C. The resulting suspension was centrifuged and dried before being placed in the pyrolysis reactor. Afterward, 3DFAB derived from green macroalgae was synthesized by pyrolysis at 700 °C for 2 h and subsequent reflux with H_2_SO_4_ and HNO_3_ (1:3 by volume) at 80 °C for 6 h.

### Synthesis of CoTLM

A suspension composed of 1 g 3DFAB, 100 mL of distilled water, Ammonium Hydroxide as pH adjusters (to set pH at ≈11), and 0.25 g of Co(NO_3_)0.6H_2_O was transferred into a Teflon-lined stainless-steel autoclave and heated at 150 °C for 15 h. The resulting products were centrifuged at 5000 rpm. CoTLM was obtained after being washed with distilled water and ethanol several times.

### Preparation of working electrodes in supercapacitor

A homogeneous mixture composed of synthesized samples (RAB, 3DFAB, or CoTLM), carbon black, graphite, and polytetrafluoroethylene (PTFE) was prepared with the assistance of a few droplets of ethanol. Afterward, the mixture was pressed on a piece of a stainless steel 316 mesh 200 current collector (0.5 cm × 1 cm) under a pressure of 10 M Pa, and the amount of active material for each electrode was ranged from 2–3 mg. To fabricate the asymmetric supercapacitor of 3DFAB//CoTLM, 3DFAB and CoTLM were assembled into an MTI cell as negative and positive electrodes, respectively. In this system, the mass ratio between positive and negative electrodes was equal. The glassy fiber (GF/A) was used as a separator and 3 M KCl was introduced as an aqueous electrolyte.

### Characterization of materials

The morphological characteristics and pore structure were investigated through field emission scanning electron microscopy FESEM (MIRA3 LM, Tuscan) at an acceleration voltage of 15.0 kV. Fourier Transform Infrared (FTIR) spectroscopy (Brand: Bruker ISS-88) was used to determine the type and intensity of attached functional groups over synthesized materials. New crystallinity phases resulting from reaction and interaction among components were determined by studying the XRD patterns recorded by Xpert MPD diffractometer. Textural properties of RAB, CoTLM, and 3DFAB were analyzed by TriStar II 3020 Version 3.02 through Brunauer Emmett Teller (BET) equation. X-ray photoelectron spectroscopy (XPS) measurement was performed in a Perkin Elmer PHI 6000C ECSA system with monochromatic Al KR (1486.6 eV) irradiation.

### Electrochemical measurement

The electrochemical parameters (CV, CCV, GCD, and EIS) of state-of-the-art electrodes were measured using a three-electrode system comprised of CoTLM (3DFAB or RAB) working electrode, an Ag/AgCl reference electrode, and a graphite rod counter electrode. We implemented the same instruction for determining CV, CCV, and GCD, but in a two-electrode system. All electrochemical data were collected using an Autolab 302 N, at 25 °C in 3 M KCl electrolyte.

As for the SC calculations of three-electrode systems using GCD curves, the following formula is applied:

9 $$ {\text{SC}} = \frac{{{\text{I}}\mathop \smallint \nolimits_{T1}^{T2} \Delta s}}{m\Delta V} $$

where SC (F g^−1^), I(A), ΔS, m (g), and ΔV (V) are referred to specific capacitance, real current discharge, integrated area of discharge curves, active mass of the single working electrode, and potential window. The equation for the asymmetric cell operates in a pretty similar fashion, but with a minor change in that the numerator is multiped by two. For 'm' average active mass of both electrodes is considered (equal mass).

## Conclusions

In summary, we have fabricated a cost-effective ASC using RAB and 3DFAB as negative electrodes and CoTLM as a positive electrode. CoTLM was synthesized by integrating pyrolysis and hydrothermal carbonization methods. XRD, FTIR, and FESEM-EDS analyses confirmed the unique hierarchical architecture and superior surface area of CoTLM, resulting in high specific capacitance and excellent cycling stability. The potential operating range of 3DFAB was − 0.9 to 0 V, while that of CoTLM was − 0.1  to  0.9 V. Thus, this two-electrode combination's cell voltage has extended up to 1.7 V, which is significantly higher than that obtained in symmetric type supercapacitors. In terms of its capacitance, it holds an SC of 411 F g^−1^ at the current density of 1 A g^−1^. Besides that, the capacitance remained unchanged after 4 k cycles at 4 A g^−1^. The energy density acquired from the 3DFAB//CoTLM ASC device is 54.44 Wh kg^−1^ with a power density of 800 W kg^−1^, which is significantly larger than that of the RAB//CoTLM (23.22 Wh kg^−1^ at a power density of 850 W kg^−1^). These values are significantly higher than those reported for cobalt and iron-based composite ASCs.

## Supplementary Information

Supplementary Information

## Abbreviations

EDLC: Electrical double-layer capacitance
ASC: Asymmetric supercapacitor cell
RAB: Raw algal biochar
CoTLM: Tile-like microstructure containing cobalt oxides
3DFAB: 3D interconnected mesopores network
SC: Specific capacitances
AAEM: Alkali and alkaline earth metals
EIS: Electrochemical impedance spectroscopy
PTFE: Polytetrafluoroethylene
CCV: Continuous cyclic voltammetry
ASC: Asymmetric supercapacitor cell
FTIR: Fourier transform infrared
XPS: X-ray photoelectron spectroscopy
FESEM: Field emission scanning electron microscopy
BET: Brunauer-Emmette-Teller
XRD: X-ray diffraction
CV: Cyclic voltammetry
GCD: Galvanostatic charge/discharge
