# Supplementary material for: Integrated hybrid architecture of metal and biochar for high performance asymmetric supercapacitors
Source: Sci Rep. 2021 Mar 8;11:5387. doi: 10.1038/s41598-021-84979-z (PMC7940490; doi:10.1038/s41598-021-84979-z)
Supplement: Supplementary file 1 — Supplementary Information [file 41598_2021_84979_MOESM1_ESM.docx]

**Integrated hybrid architecture of metal and biochar for high performance asymmetric supercapacitors**

**Omid Norouzi^1,4^,** **S.E.M Pourhosseini^2,3^, Hamid Reza Naderi^3^, Francesco Di Maria^4^, Animesh Dutta^1*^**

^1^School of Engineering, University of Guelph, Guelph, N1G2W1, Ontario, Canada

^2^Institute of Chemistry and Technical Electrochemistry, Poznan University of Technology, 60-965, Poznan, Poland

^3^School of Chemistry, College of Science, University of Tehran, Tehran, Iran

^4^ Department of Engineering, University of Perugia, Via G. Duranti 67, 06125 Perugia, Italy

E-mail: [adutta@uoguelph.ca](mailto:adutta@uoguelph.ca). Tel.: +1 519-824-4120 ext: 52441.

First author E-mail: [Norouzio@uoguelph.ca](mailto:Norouzio@uoguelph.ca)

**Figure. S1. FESME images.**


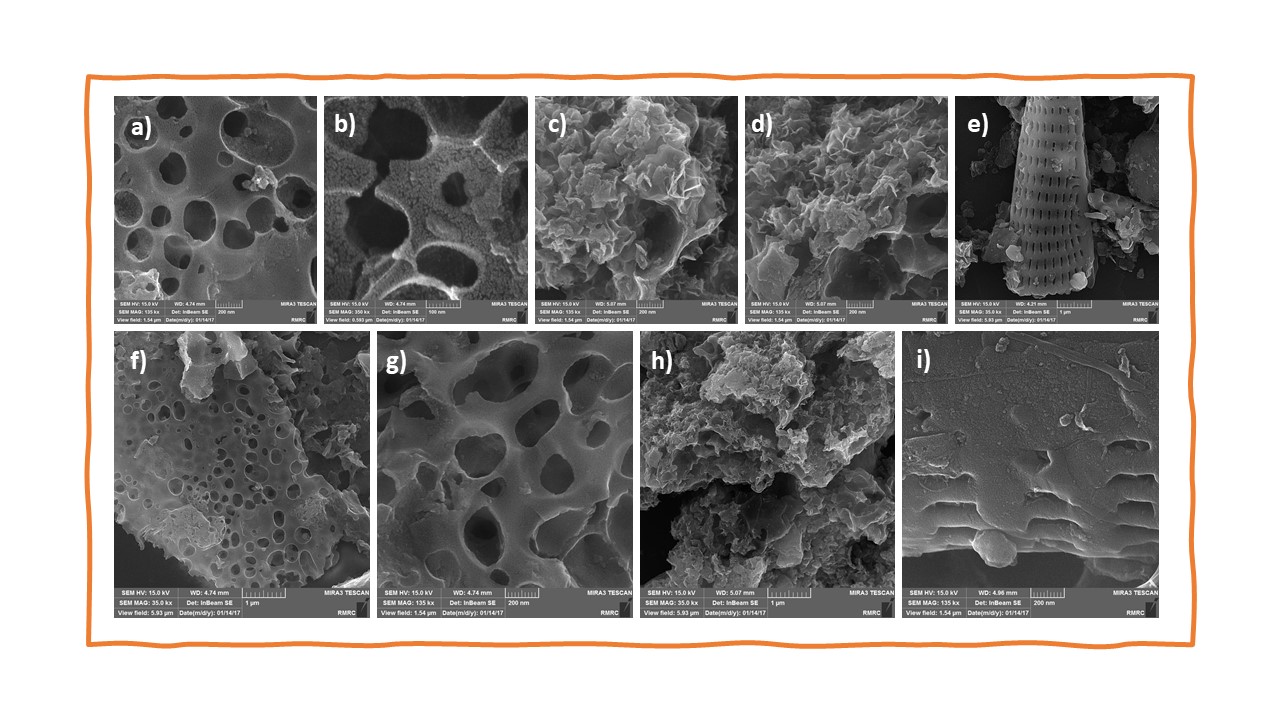


Fig. S1 (a,b,f, and g): 3D interconnected mesopores network

Fig. S1 (c,d, and h): Tile-like Microstructure containing Cobalt oxides

Fig S1 (e, and i): Raw Algal Biochar
